# Ethical sharing of health data in online platforms – which values should be considered?

**DOI:** 10.1186/s40504-017-0060-z

**Published:** 2017-08-21

**Authors:** Brígida Riso, Aaro Tupasela, Danya F. Vears, Heike Felzmann, Julian Cockbain, Michele Loi, Nana C. H. Kongsholm, Silvia Zullo, Vojin Rakic

**Affiliations:** 1 Instituto Universitário de Lisboa (ISCTE-IUL), Edifício ISCTE, Av. das Forças Armadas, 1649-026 Lisboa, Portugal; 2 Department of Public Health, University of Copenhagen, Copenhagen, Denmark; 3 Centre for Biomedical Ethics and Law, Department of Public Health and Primary Care, KU Leuven, Leuven, Belgium; 4 Leuven Institute for Human Genetics and Society, Leuven, Belgium; 5 Centre of Bioethical Research and Analysis, Philosophy, School of Humanities, NUI Galway, Galway, Ireland; 6 University of Gent, Ghent, Belgium; 7 Institute of Biomedical Ethics and History of Medicine, University of Zurich, Zurich, Switzerland; 8 ETH Zürich, Department of Biology, Institute of Molecular Systems Biology, Zürich, Switzerland; 9 Section of Philosophy, University of Copenhagen, Copenhagen, Denmark; 10 Department of Legal Studies, CIRSFID, University of Bologna, Bologna, Italy; 11 Centre for the Study of Bioethics, University of Belgrade, Belgrade, Serbia

**Keywords:** Data sharing, Ethical values, Health data, Health research, Information and communication technology platforms, Interoperability

## Abstract

Intensified and extensive data production and data storage are characteristics of contemporary western societies. Health data sharing is increasing with the growth of Information and Communication Technology (ICT) platforms devoted to the collection of personal health and genomic data. However, the sensitive and personal nature of health data poses ethical challenges when data is disclosed and shared even if for scientific research purposes.

With this in mind, the Science and Values Working Group of the COST Action CHIP ME ‘Citizen's Health through public-private Initiatives: Public health, Market and Ethical perspectives’ (IS 1303) identified six core values they considered to be essential for the ethical sharing of health data using ICT platforms. We believe that using this ethical framework will promote respectful scientific practices in order to maintain individuals’ trust in research.

We use these values to analyse five ICT platforms and explore how emerging data sharing platforms are reconfiguring the data sharing experience from a range of perspectives. We discuss which types of values, rights and responsibilities they entail and enshrine within their philosophy or outlook on what it means to share personal health information. Through this discussion we address issues of the design and the development process of personal health data and patient-oriented infrastructures, as well as new forms of technologically-mediated empowerment.

## Introduction

During the past decade the developments in platforms for data sharing have increased considerably and calls to share data have intensified (Arzberger et al. 2004). Some have argued that our societies have become both data rich and data dependent in that the proliferation of data producing sources has increased exponentially, along with our need to use and analyse increasing amounts of data (Rodriguez 2013). This has given rise to the notion of ‘big data’ as a major driver of research and development within the biomedical sector (Leonelli 2014). From a policy perspective, data sharing has become a preoccupation with all major policy actors and supranational organisations seeking to develop and bolster data generation and sharing strategies (OECD 2009; Laurie 2004; European Society of Human Genetics (ESHG), 2003). In addition, a massive increase in the uptake of electronic health records has made access to and analysis of massive data sets a reality (Murdoch and Detsky 2013). Numerous actors, ranging from national health authorities to businesses, as well as academic researchers, have been developing new types of information infrastructures that can facilitate the sharing of personal health data (Bansler and Kensing 2010; Gherardi et al. 2014). Scandinavian countries in particular have a long-standing policy of utilising their populations for medical research, capitalising on the pervasive use of social security numbers to track individuals through a multitude of registries and databases (Hoeyer 2016; Bauer 2014).

In this respect, the so-called knowledge-based society visions suggested by the European Union (EU) and the European Commission have come to represent a new form of civil society (European Commission 2005; Felt and Wynne 2007). At the same time, some authors have argued that the demand and interest in collecting and analysing more data from more people has led to a type of data fetishization (Lupton 2014). Models of knowledge production and use enshrine specific values, which are reflected in the types of platforms that are developed for sharing data. Some authors, such as Epstein (1995), Rabeharisoa and Callon (2002) and Novas (2007) have suggested that patient-led activism has become an important driver within the biomedical research community, accounting for not only the mobilisation of patients as research participants, but also in raising capital for research itself (see also Rabeharisoa et al. 2014). In this sense, patient activism is being viewed as a novel form of social movement, which is, in part, driving the demand, and creation of new platforms for the generation and distribution of data (Tupasela et al. 2015). Lupton (2014) argues that digital media platforms which elicit lay people’s experiences of illness and healthcare are giving rise to ‘the digital patient experience economy’ where not only are patients able to express their experiences in more diverse ways, but are at the same time being exploited using novel strategies, for both research and commercial purposes. This process is situated within a broader context in which big data is valorised through a discourse of data sharing not always clear in its intentions (Van Djick and Poell 2016).

In this article, we seek to examine five data sharing platforms (Taltioni, Healthbank, MIDATA, ePGA, and PEER Network) that have been developed to facilitate the sharing of personal health data within a variety of scenarios. We argue that these platforms reflect diverse conceptions of data sharing among stakeholders, as well as specific types of core values associated with data sharing. The relationship between data sharing and the platforms that facilitate this are therefore a crucial issue in understanding the role and significance that different data sharing initiatives seek to achieve. We argue that data sharing is not a value-neutral practice, but rather represents a broad spectrum of ethical, political and social goals that various actors are seeking to achieve. By identifying core values, which exist in sharing platforms, we seek to develop a general typology of basic principles of operation that we considered essential for an ethical sharing of health data.

In the following, we first discuss some of the general challenges related to sharing personal health data. We then outline and describe in detail the core values we have identified. Subsequently, we present and discuss five data sharing platforms as cases studies in order to illustrate whether or not these core values are realised by existing platforms before discussing the significance of these cases in relation to these core values.

## Health data sharing: An interoperability policy in the making

Many public and private funding agencies recognise the value of shared data. However, if data sharing is to be promoted, reciprocity and societal incentives play a significant role (Piwowar et al. 2008; Gottweis et al., 2011). Data sharing data is a form of cooperation and it presupposes some notion of mutual advantage. If a platform for data sharing is perceived to only promote the benefits of the platform owners, or their business partners, it will not invite significant participation from the population. The mutual benefit provided to platform participants does not have to consist in money or some other individual advantage. It could also consist - as it does in some of the models we will explore in what follows - in providing participants with a new opportunity to act altruistically*.* Using Amartya Sen’s distinction between *agency* and *well-being* (Sen 1985), it could be said that the benefit provided by the platform to the users may also consist in an expansion of their *agency*, even when it does not directly contribute to their personal well-being*.* As we shall see in the section on the value analysis, a broader notion of reciprocity, which considers all participants in society as (indirect) contributors and beneficiaries to data sharing, is also pertinent.

Data sharing has in some cases taken on values related to notions of the general public good, such as with blood donation in which donating to a common good is seen to produce social rewards (Titmuss 1970). As Prainsack and Buyx (2017: 106) have suggested, contributing to a database can generate social value. Still, data sharing in biomedical field has some real obstacles in relation to developing common standards and platforms for sharing in order to make sharing more meaningful and effective. Society can, for example, facilitate reciprocity by providing infrastructure as platforms with proper safeguards, making it easy to access and share data while protecting both scientists and donors. This could be achievable by requiring platforms to demonstrate how their endeavours contribute to the common good – for instance, by allowing anonymised data to be exported to public institutions for public health purposes. Also, by requiring platforms to provide a high degree of autonomous control of the provided personal data to the their user, society can promote reciprocity. Since the platform users’ and owners’ interests are not always aligned, enhancing user-centric control is expected to enable the users to reappropriate at least some benefits from the data.

Platforms can be viewed as a regulated environment enabling developers, users, and others to interact and share data, services, and applications, while also enabling governments to support the development of innovative solutions. But platforms are not silos: they need to be integrated with other platforms and systems, while ensuring their interoperability, an effort that may prove more controversial than initially thought.

When discussing data sharing, the term ‘platform’ requires explanation. Keating and Cambrosio (2003: 27) have suggested that the term platform can be seen as a semantic spectrum where, at one end you have an engineering/physics concept of a bench onto which other devices can be attached. At the other end of the spectrum, you have the political platform, which refers to an arrangement of statements and positions in relation to particular issues, whereby the platform refers more to a way of organising and arranging activities, as well as material (including data). The spectrum, however, points our attention towards the organisation of activities that have very pragmatic goals of making various activities work. At the same time, platforms represent the enshrinement of values and expectations of what data sharing will achieve.

While governments and authorities are calling for a new approach to the sharing of data and are developing policies for data sharing platforms, they have not yet adequately addressed the obstacles that underpin the failure to share data (Pearce and Smith 2011). Nor are they sufficiently recognising the emergence of diverse systems for facilitating sharing (Thilakanathan et al. 2014). For example, each European Union (EU) country has its own rules and codes of practice, and these pose an obstacle to effective data sharing across national boundaries. Without an integrated model or agreement on the core values enshrined in sharing, we cannot achieve logistical fluency (Mascalzoni et al. 2014).

The sharing of data can be implemented on different levels: within an organisation, between organisations, across national borders, or between healthcare institutions and citizens. Each of these contexts carries with it its own specific challenges. E-Health systems are adopted by end-users and have the ability to interface with national and international health systems. This has an impact on the roles and liability of stakeholders, and on patients’ ability to give informed consent for personal data processing (Dunlop 2007). Cross-border and interoperable electronic health-record systems make confidential data more easily and more rapidly accessible to a wider audience. However, by enabling greater access to a compilation of personal data concerning one’s health and genetic information from different sources, and spanning a lifetime, they increase the risk that personal health data could accidentally be disclosed or distributed to unauthorised parties (Hoffman 2010). There is broad agreement that it is individuals who should not only control their own data but also have the right to make decisions about access to their data, and be informed about how they will be used (Kaye et al. 2011; Brent D Mittelstadt et al. 2012; Solove 2013; Sterckx et al. 2015). Nevertheless, control over their own data implies also the possibility of withdrawing the information, which in some cases, could not be guaranteed since data sharing through these platforms tend not only to combine different kinds of data but also to share them through complex networks (Shabani and Borry 2015).

Data storage and regulations for processing personal data raise several concerns about how that data should be used. This should entail a balancing of individual rights and interests against social benefits. Such an approach presumes value judgment in favour of individual control over highly beneficial uses of data. Still, that value choice turns out to be problematic when it comes to balancing the principle of privacy and data protection against other societal values such as public health, national security, environmental protection, and economy efficiency.

Tene and Polonetsky (2012) have argued that a coherent framework would be based on a risk matrix, taking into account the value of different uses of data against the potential risks to individual autonomy and privacy. If public health is a common good from which everyone benefits, and is essential to human development, then in the current debate on building a data sharing environment we need to address the important issue of how health data can be used for the common good, while still respecting individual rights and interests, such as the right to privacy. Yet, we also need to determine which trade offs are acceptable between individual rights and the common good, and how the thresholds for such trade offs can be determined.

It is within this broader context that we are interested in the ways in which emerging data sharing platforms are reconfiguring the data sharing experience from different perspectives. What novel forms of data sharing are made available? What do they allow? And what types of values, rights and responsibilities do they entail and enshrine within their philosophy on what it means to share data?

## A framework for ethical sharing of health data in online platforms

Moral values are an indispensable component of successful mechanisms of data sharing. Gaining clarity about the values at play is crucial before we can determine which values need to be retained and which may need to be traded off against each other. Identifying the core values required for morally responsible data sharing also highlights how the development of technologies is not value-free, but always reflects and enshrines particular beliefs (Langat et al. 2011).

In this respect, we identified six core values that we considered to be essential for the ethical sharing of data using ICT platforms: scientific value, user protection, facilitating user agency, trustworthiness, benefit and sustainability. To do this, we utilised the experience and expertise of members of the Science and Values Working Group of the COST Action CHIP ME.^1^ This was an iterative process where an initial list of aspects considered important for ethical data sharing more broadly were brainstormed by members of the working group, many of which have considerable experience with ICT platforms. Moreover, we acknowledge that listing values entails already a process of valuation, on our behalf, and constitutes per se a performative process. The discussion was then shifted to focus on aspects specific to ICT-based platforms and how these aspects could be applied in this setting. The final list was refined through discussion, grouping of concepts to avoid redundancy, and feedback from other working group members not present at the initial discussion. Before undertaking our exploration, we were aware that others had already considered the principles and values to be respected in the sharing of personal health data. For example, Kelty and Panofsky (2014) in their assessment of participation in online platforms and Prainsack (2014a) in her typology of citizen science initiatives, for example, have touched upon the issues of data sharing and both provide critical insights on data sharing and user’s experience. However, in our opinion, both scenarios benefit from a proper ethical reflection focused on data sharing, since that is not their aim. In this sense, the Global Alliance for Genomics and Health (GA4GH), for example, answers this purpose. GA4GH has set out on its website the following eight foundational principles (Global Alliance for Genomics and Health 2014): 1) *respect* for the data sharing and privacy preferences of participants; 2) *transparency* of governance and operations; 3) *accountability* to best practices in technology, ethics, and public outreach; 4) *inclusivity* by partnering and building trust among stakeholders; 5) *collaboration* to share data and information to advance human health; 6) *innovation* in order to develop an ecosystem that accelerates progress; 7) *agility* to ensure we act swiftly to benefit those suffering with disease; 8) *independence* by structure and governance.

However, to us, these principles are somewhat vague and require further in-depth consideration of the underlying problems, which surface through the sharing of health data. For this reason, in our analysis we set aside the GA4GH principles in an attempt to develop a more detailed list of core values that we considered to be important factors for the ethical sharing of data using ICT platforms.

While we recognise that there is some overlap between the values, our intention was to create ideal categories through which an analysis of various platforms could be conducted. The six core values are described in detail below.

### Scientific value

When discussing scientific value in online data sharing platforms, we are referring to the need for quality, quantity and accessibility of data to be of sufficient standard to allow morally responsible data sharing. Data quality concerns parameters such as accuracy, reliability, completeness and consistency (OECD 2013, Rippen and Risk 2000). It also refers to the introduction of bias in the dataset by means of the ways the data are collected (Vayena and Tasioulas 2013, Leonelli 2014). Data quantity acquisition relates to the ability to collect and connect large datasets. We need a certain amount of data for them to be useful: a greater data set may support inferences that a smaller data set does not support (Mayer-Schönberger and Cukier 2014). Still, a trade-off may be necessary between data quality and data quantity. A platform may enable the collection of large quantities of data but, due to the large scale, the data quality may be less accurate, reliable, complete, and consistent.

Another contributor to the scientific value of a platform is data accessibility, which depends on the interoperability of health data sets: the ability of different health data systems to exchange information accurately and to use the information that has been exchanged (Heubusch 2006). Open standards and interfaces are useful to enable a broader utilisation of information, potentially by all stakeholders that may derive a benefit from them. It is also important to note that there will be a trade off between data accessibility and user protection, because the greater the opportunities for sharing data, the greater the opportunities for health data to be misused.

### User protection

While the term user protection is often used in relation to privacy, in the context of morally responsible ICT based health data sharing; we consider that user protection could also include the protection of user’s dignity, the protection of user’s confidentiality, data security, and informed consent.

Privacy is related to the value of autonomy. Even if our privacy is being invaded without consequences that we consider to be detrimental to us, the very fact that we have not been asked infringes our autonomy. As a result, levels of trust in healthcare system might decrease. The value of privacy is also linked to dignity: the fundamental right of every person to be valued, respected and be treated in a morally appropriate way (Global Network 2015). If our privacy is being invaded, our dignity might be brought into question.

Moreover, privacy as control of information is connected to informed consent, the “[a]greement to a certain course of action, such as treatment or participation in research, on the basis of complete and relevant information by a competent individual without coercion” (Global Network 2015: 29). Informed consent is intended to prevent the unauthorised usage of a participant’s own data in ways that are unknown, poorly understood, or in conflict with the values and commitments of the data donor.

### Facilitating user agency

Facilitating user agency implies a promotion of autonomy – the capacity of an individual to be self-governing, make decisions, and to act in accordance with them, as well as with her values, commitments and life goals (Global Network 2015). Enhanced user agency is often associated with empowerment and is promoted when platforms provide relevant features to their users. These features include communication, education, and informed consent. Others should not be authorised to do things to us, for us, or in the name of us, in ways that are inconsistent with our values, goals, opinions, and life plan. One relevant question is whether our autonomy is violated if our genetic data or biospecimens are being used without our consent to help others (e.g., to help people suffering from rare diseases). However, autonomy may not always be the most important value (Dawson 2010): it might be argued, for instance, that when the physical and privacy risks for participants are negligible, proper safeguards are in place (such as anonymisation), and data are being used to promote public health (or other public goods), even the re-use of data or biospecimens for purposes other than those for which they have been initially collected might be considered as morally appropriate (Global Network 2015). Moreover, various surveys show that the vast majority of people are willing to make their genetic data and biospecimens available, provided that their permission to do so is sought (Wellcome Trust 2013).

### Trustworthiness

Trustworthiness is a feature of human relations that “is needed precisely when and because we lack certainty about others’ future action: it is redundant when action or outcomes are guaranteed” (O’Neill 2002: 13).

It appears that reliance on informed consent and other measures to secure individual autonomy have failed to secure trust (O’Neill 2002; Chadwick and Berg 2001). Some clumsy approaches within the Care.data scheme in the UK highlighted the importance of trust and the effort which should be invested in keeping the trust of citizens intact when the acquisition of their health data is concerned (Sterckx et al. 2015). Hence, trust is an essential and indispensable value for morally apposite sharing of health data. It is tightly connected to the values of transparency, the accessibility of information about management and its decisions, and accountability - the existence of clear procedures and a clear division of responsibility in responding to challenges related to health data sharing (Global Network 2015).

As platforms are run using information technology, one important aspect of transparency is associated with the code behind these platforms, which can be inaccessible proprietary software or, more transparently, open source software where the published code is publicly available. The publication of a platform source code in an easily accessible online repository means that any user can, in principle, examine the software to determine if the claims made by platform managers about the way privacy is handled correspond to the facts. Finally, trust is connected with the capacity of an institution to provide some sort of compensation or protection to parties that are harmed (either by accident or due to negligence) (Prainsack and Buyx 2013).

### Benefit

We contend that equity in the distribution of benefits is an important value related to morally appropriate sharing of health data. We use the concept of benefit as a wide umbrella term to cover all issues relevant to the *augmentation* of benefits and their *distribution,* or, in other words, to the value of *efficiency* and *distributive justice*. We can make our genetic data or biospecimens available for research and serve a common goal in that way. But such actions might also serve our own interests, in that we may ourselves (as individuals) reap the benefits of discoveries in the field of medicine. To ensure a fair balance of interests, proper mechanisms for benefit sharing should be put in place when designing data sharing platforms (Chadwick and Berg 2001).

Data sharing may also entail broader societal goals, which align more with the notion of reciprocity, where the benefits produced by the data should be shared among all parties involved in the production of that data (platform users and owners). According to the theory of justice of Rawls (1971), since the production of goods relies on well functioning *social institutions* the distribution of advantages produced within social institutions is always embedded in relations of reciprocity with all the citizens who are responsible for upholding and sustaining social institutions, so relationships of reciprocity are quite broad, and reach out to persons who are not directly involved in the generation of data.

It has been argued that every conception of justice relies on a specific interpretation of the concept of equality: even libertarianism can be considered as a theory advocating equality of negative rights, and utilitarianism as a theory advocating equality of marginal utility (Sen 1995). However, with equality understood as equality of welfare, opportunity for welfare, or access to advantage (Arneson 1989, Cohen 1989), there will normally be trade offs between equality and efficiency.

One issue of justice in relation to benefit sharing has to do with access to the benefits of research by disadvantaged groups and populations. Their members may be put at risk by participation (e.g. by virtue of privacy loss) while they may not be able to benefit from the knowledge generated. For example, intellectual property over new drugs and other technology provides incentives for research and development, but also excludes disadvantaged members from the benefits due to the price of new drugs and services, which may not be covered by universal healthcare services and insurance systems (Global Network 2015). As such, both the amount of benefits and their distribution are important moral considerations and there is no consensus about the optimal balance between them. Judgments about the justice of the sharing of benefits generated by a certain platform should take this into account (Tang et al. 2006). Issues of benefit sharing also emerge in the selection of topics for research (Global Network 2015).

### Sustainability

In the context of morally responsible data sharing, sustainability refers to the expectation that the platform will continue to deliver its services for a sufficient period of time, thus justifying the costs (economic and other) of building it, or that the platform terminates operations promptly on failure. Typically, sustainability is achieved by means of a realistic business plan, specifying where the long term resources that are required come from and what will happen to the informational resources collected or generated on termination or transfer of ownership or control of the platform. One aspect of sustainability concerns the universality of a platform’s business model. Being more dependent on idiosyncratic cultural or financial circumstances, that cannot be reproduced in other countries or as political circumstances change, can be viewed as a weakness. In the world we live in, a sustainable platform should not rely exclusively on ad hoc public funds or the benevolence of charitable donors but significantly, on the revenues it generates by providing wider benefits to an entire research and industry ecosystem (Harris et al. 2012).

Sustainability is closely linked to issues of benefit and fairness and again, there may a need for trade offs between different desired features. For example, a platform may be operated as a ‘for profit’ business entity, have a realistic business plan for generating a flow of revenues and attract private investment for its initial funding. Yet, because of the role of private investors, it may have to channel most benefits to, or be controlled by, its majority shareholders. This could in turn negatively affect transparency, benefit sharing, and promotion of user agency, since the people who use the platform to share their data may have little opportunity to voice criticism about and effect the economic choices of its management. On the other hand, a model for a platform could fulfil the ideal criteria of transparency, user empowerment and benefit sharing, and then not be feasible, let alone sustainable, because it is not perceived as an attractive investment by those entities (public or private) that can fund it.

## Translating ethical values into practice: Five examples of online platforms for health data sharing

In order to assess how well these core values were addressed by online platforms for health data sharing, purposive sampling was used to identify examples of data sharing platforms as case studies representing a broad range of potential uses, situations and challenges.

Selection of platforms was restricted to those orientated for health data sharing. The specific sampling categories used to identify these data sharing platforms were as follows: 1) sector (public, private, or public-private initiatives), 2) primary goal (health research or health data sharing), 3) country specificity (national and cross-national), 4) platform leadership (patient-led or researcher-led approach). The broader working group was also asked to highlight platforms with the following specific features of interest: 5) large scale data sharing, 6) platforms which allow sharing of genetic information, 7) platforms with some participatory features (Web 2.0 or other), and 8) distinctive/innovative ICT solutions, approaches or functionalities.

In our primary search for platforms we identified numerous examples that sought to collect and share personal health data and information of different types. We have listed these examples in Appendix. We subsequently selected five platforms in an effort to illustrate a range of different perspectives and diverse models of action. Table 1 shows an overview the main characteristics of each selected platform.

**Table 1:** Features of the selected online platforms for sharing of health data

Platform entity	Taltioni	Healthbank	MIDATA	ePGA	PEER
Scope	National (Finland)	International (Based in Switzerland).	National (Based in Switzerland)	International (based in Greece).	International (based in United States)
Sector	Public-private. Finnish government; Member organisations (public and private).	Private. Investments of partners and members/users.	Public –private. Research grants, loans.	Public. Public grants (EU and Greece).	Public-private. Public and private funds, Crowdsourcing.
Primary goal	Health data sharing. Health data safe storage and management; well-being apps.	Health data sharing and health research. Safe storage; research; user initiated selling of health data.	Health data sharing and health research. Data safe storage and management; research; data sharing; third-party services (for-profit) on data made accessible by members.	Health research. Genome-based recommendations about gene-drug-phenotype interactions open access pharmacogenomics data.	Health data sharing and health research. Biospecimen sharing. Joining communities within the PEER platform.
Leadership	Users and researchers-led.	Users and researcher-led.	Users and researchers-led.	Researcher-led.	Patient-led.
Data types	Health and lifestyle related data.	Health and lifestyle related data.	All personal data.	Established genomic databases; Genomic profiles and phenotypes uploaded by users.	Health related data.
Large scale data sharing	No.	Yes.	Yes.	Yes.	Yes.
Participatory features	Users can decide which data sets to share and with whom.	Users decide which data sets to share and with whom. The data available is uploaded by the users.	Users decide which data sets to share. Users can give specific third- parties access to run analyses on their data on the platform.	Data is uploaded by the users.	Users decide which data sets to share and with whom.
ICT innovative solutions	Focus on personalisation: tailor usage according to individual needs.	Works like a bank: users invest their data and collect the profit from their usage in research.	Enables the combination of health data with other personal data. It is possible to release all the data to researchers.	Orientated for supporting health professionals to increase their knowledge in genomics and to support them in providing appropriate healthcare.	Enrolment on patient networks while allowing contributions for accelerating health research.

Here we present these five case studies to highlight examples of how these six core values are addressed by online data sharing platforms. Information on these platforms was gathered from their websites, published materials on the platforms and, in some cases, interviews or email contact with persons associated with these platforms. Although these five platforms address the core values in different ways and varying degrees, they represent models for data sharing with innovative features in relation to how data is shared between different stakeholders. Table 2 summarises how the core values are addressed by each platform.

**Table 2:** Overview of how values are addressed by each platform

Platform entity	Taltioni	Healthbank	MIDATA	ePGA	PEER
Scientific value	Data quantity and quality is determined by the users who upload data.	Data quantity and quality is determined by the users who upload data.	Data quantity and quality is determined by the users who upload data.	Established genomic databases; Genomic profiles and phenotypes uploaded by users.	Data quantity and quality is determined by the users data upload.
User protection	Privacy policy. Require users’ consent to share general data. Require users’ consent to share specific set of data.	Privacy policy. Require users’ consent for all sharable data.	Strong encryption and data access log. Only users can access their data.	Password protected profile. No information about security features yet.	Privacy policy. Users receive a system report with all the system changes (edits, data usage, etc.).
Facilitating user agency	Users are responsible for choosing apps of interest and for the data uploaded.	Users decide whether or not to share their data and to which research studies. Members can participate in cooperative decisions.	Users decide whether or not to share their data and to which research studies. Members can participate in cooperative decisions.	Features stressing user agency might be included in the future (the platform is still underdeveloped).	Users decide whether or not to share their data and to which research studies. Users can also decide who is going to see the data. Possibility of joining patient communities.
Trustworthiness	The services have to pass Taltioni’s audit. Disclose which data is going to be shared and with whom.	Each use of data has to be disclosed to the users.	Platform IT code is open source. Every research project will need ethical committee approval.	Developed within public sector (which might increase trust). No other relevant information is provided.	The familiar environment promoted by patient communities ensures trust in the platform.
Benefit	Users can choose apps of their own interest and receive self-help and well-being services. ICT companies can offer paid services and develop another apps and services.	Profit (in cash or not) is divided between participant users. Private investors could also benefit (in cash or not).	Upgrade of the services provided by the platform.	Patients may benefit from different health care from the increased knowledge of health professionals in genomics.	Users can benefit from networking engagement. Researchers benefit from using data.
Sustainability (Funding)	Cooperative of Finnish government and ICT companies.	Cooperative and associated commercial company. Investments of partners and members/users.	Users fees (users and end-users such researchers); research grants, citizen loans.	Public national and international grants.	Public and private funds and crowdsourcing.

### Taltioni

The Taltioni platform is a national data sharing platform in Finland. The platform is provided by the Taltioni Cooperative which involves both the public and private sector. It focuses on health status control, health management, health promotion and well-being. As a national platform, access to Taltioni and its range of features requires a Finnish social security number, which allows it to be linked with various personal data records. Although some sections of the website are available in English, many documents, such as the data sharing policy are only available in Finnish.^2^

The platform is organised as a toolkit, including applications for smartphones and mobile devices, and allows users to download a selection of free applications (or ‘apps’) and to tailor them to the individual’s needs. The users can choose among a wide range of apps, from blood pressure record, weight management or fitness, to organisation of the patient’s medication schedule, management of medical appointments or access to their own health data. In addition, the platform connects the user and the healthcare system in both directions, providing practical services for users based on their existing health information, and also enabling the upload of data about the individual’s health and well-being. Taltioni makes use of an innovative way of sharing personal health data since it harnesses the potential of mobile devices, allowing it to be accessed from any location with Internet access.

The Taltioni toolkit structure facilitates user agency: through their ability to utilise applications for their own purposes, users can store their own data and share with friends and family, facilitating the creation of close networks and virtual communities. These features could also contribute to benefit individual users. In fact, the strong focus on personalisation, particularly for users to tailor their usage according to their individual needs, is the other distinctive feature of Taltioni. Still, long-term benefits depend on the type of functionality that will be offered through the third-party programs that people will have access to.

User protection is addressed through both the security features of the platform and the privacy policy. Taltioni uses two different types of consent regarding two sets of data, which are directly related to the platform structure: an umbrella platform that hosts apps from different providers. In this sense, users have to consent in sharing general personal information needed to open a Taltioni account (e.g. name, address, social security number) and to give an additional consent related to the type of application the user will share their health data with (the user will be asked to give specific consent for such use within each subscribed application). As a way to ensure trustworthiness Taltioni in its Data Protection Agreement disclose the kind of information that is going to be collected and stored in the platform (name, email address, language, citizenship). Taltioni also states that information such as identification number and name are removed for statistical purposes, allowing third parties and companies to use anonymised data. All other information is shared with service providers under specific user agreements that might vary among the different apps. Furthermore, data cannot be transferred outside of the EU. The users decide which information they wish to share with the provider.

However, the personalisation enabled by Taltioni has also a flipside. The platform is portrayed as a method for Finnish people to take responsibility for their own health, both in the media and in the platform’s self-representation (cf. Turun Sanomat 2013; Sitra, 2012). This idea corresponds to a general trend toward shifting the focus of health-related responsibility from government and healthcare systems to individuals (Prainsack 2014b). Plus, the personalised nature of the features offered by Taltioni could make the creation of standardised information or a nuclear set of information difficult which, of course, could compromise the scientific value. In addition, the diversity of companies developing apps and promoted them through the umbrella platform could turn the development of data standards even more complex.

The cooperative nature of Taltioni promotes the sustainability. The platform is run and maintained by a number of ICT companies who work together to provide other companies with the possibility to develop sharing platforms through Taltioni. In fact, the enterprises behind Taltioni could offer some paid services through their free apps or use the user’s records for developing new services. As a consequence, ICT companies would be stimulated to develop services that share common standards and practices, while ensures broad support from the industry. Whether or not Taltioni cooperative vision is going to work in practice remains to be seen, since Taltioni platform is not until now, totally functional.

### Healthbank

Healthbank is an international platform based in Switzerland. Healthbank combines a range of different services and features for health and lifestyle data storage (e.g. sleep pattern, fitness tracking) and health research. The platform is open to individuals worldwide, and can be used by researchers in any location, enabling large scale data sharing.

The platform offers a innovative way of operating. This is not only due to the wide range of data that is possible to manage and store, but also in that it offers a way for users to profit from data storage. In fact, Healthbank tries to reproduce the workflow of a traditional financial institution, as it is emphasised in the platform name. Users who want to be members pay a fee for depositing their personal information. Data can be sold to researchers and profits are divided between the participant users. The process may also be profitable for Healthbank as a whole and its private investors, attracted through the Healthbank Innovation Company (Healthbank Innovation Ad^3^). The benefits may or may not be in cash, depending on the research project. These benefits are also tied to the quality and quantity of data provided by users – a strategy that could encourage users to provide more and higher quality data.

One of primary targets of Healthbank is health research. For this reason, data quality has to meet particularly high standards. Nevertheless, data quantity and quality are still directly dependent on the amount and the type of data uploaded by the platform users. There might be doubts as to whether the introduction of economic incentives for data compromises data quality. Studies on blood donation have shown that in countries economic incentives were introduced, blood quality decreased (Titmuss 1970).

Additionally, the users are responsible for setting which data and to what extent can be shared, with whom and in which kinds of research. In doing this, Healthbank improves user agency and trustworthiness. Even though, the users’ agency could be maximised in doing this, the scientific value could, again, be compromised.

Furthermore, Healthbank makes use of other tools that enforce the values of user agency and trustworthiness: users/members are allowed to participate in cooperative decisions through a general assembly. Users can decide whether or not to share their data and in which studies they are willing to participate. They also have the option to share their records with health providers and friends. A consent manager is provided for all uploaded data and users are required to give consent for their data to be used for each research project, while each use of their data should be disclosed to them.

Healthbank seems highly committed with user protection. The platform states, in its privacy and policy statements, the platform is using encryption, highlighting that the website does not use cookies. The platform stresses that it might be possible for other companies they work with, such as Youtube or Twitter, to use traceable links and warns users not to use the links provided in order to maintain a maximum level of privacy.

Regarding sustainability, the platform is managed by a private fund in collaboration with partners from the Information and Technology (IT) sector, innovation consultants, business partners, universities and research units. Even this aspect is coherent with their financial-like structure. Although, Healthbank is organised as a cooperative, member companies can buy economic shares in the cooperative with no voting rights attached.^4^ This allows the Cooperative to attract funds from private investors that can finance the operations, as well as the development of the platform.

### MIDATA

MIDATA is a Swiss ‘citizen owned’ data cooperative. The platform enables the safe storing and controlled access to members’ data by specific authorised data users, such as researchers and pharmaceutical companies. This is not limited to health data, but includes all personal data, such as consumption, fitness, geolocation, and financial data. Data generated with mobile devices can also be directly imported into the platform.

As the other platforms assessed, scientific value is dependent on the user’s decisions of sharing data. Still, MIDATA has the potential to connect data that was collected previously and has been stored in different silos from different domains while treating the individual data subject as the only person with the moral right to authorise such linkage (Hafen et al. 2014), which could be considered a distinctive feature of this platform.

This platform addresses user protection through multiple encryptions of individual personal data sets and only members possess the key to their own data. This data architecture promotes data security and privacy, as no one, including members of the management team and other scientific partners, can access members’ data unless explicitly authorised by the user to do so. The security of MIDATA platform has been tested by three independent companies, including two teams of ethical hackers (Ernst Hafen, personal communication, 17 May 2016).

MIDATA is one of the platforms that addresses the privacy and security concerns by all the possible means. Plus, making available the open code software is a way of achieving transparency, what is not usually done by the ones in charge of collecting and distributing data (Van Djick and Poell 2016).

MIDATA is intended to enable any person, independently of their geolocation, to share sets of their data for scientific research and personalised services. The MIDATA model allows the construction of regional or national data cooperatives, based on locally binding cooperative law. Additionally, the widespread similarities between principles of cooperative governance in different countries and the adoption of similar bylaws by different national MIDATAs will enable global research projects within a shared trusted environment (Hafen et al. 2014).

Trustworthiness is addressed through the fact that MIDATA is ‘citizen owned’, meaning that the data secure storage and sharing platform is owned by persons who are also its users, organised as a cooperative of data providers.^5^ MIDATA promotes transparency since the source code of the IT platform is open source. MIDATA emphasises the role of ethical committees as a way for promoting trust. All researchers’ requests have to be approved or by a relevant ethics board or by a MIDATA ethics board, a yet to be implemented cooperative function (Ernst Hafen, personal communication, 17 May 2016).

MIDATA promotes two levels of user agency. One level is individual and relates to privacy as control of information and individual autonomy. The platform includes software tools allowing individual members to make specific subsets of their data accessible to specific end-users (e.g., doctors, family members, friends, researchers, pharmaceutical companies)^5^ (Hafen et al. 2014). A second level is collective and derives from membership in the cooperative that own the platform for data exchange. The synthesis between agency as individual freedom in sharing data and agency based on cooperative membership is achieved through a ‘constitutional’ governance model: the cooperative bylaws are approved by the majority of members and establish a framework of rules binding for all; individual users are permitted to engage with all sorts of data exchanges that are not explicitly prohibited by these common rules (approved by all members) and that are authorised by an ethics review board^4^. Members can also decide how surplus revenues of the cooperative, if any, are to be spent (e.g., for research, information, education), although the exact procedures for these options have yet to be decided^5^ (Hafen et al. 2014).

User benefit is achieved for the collective, as the cooperative as a whole can benefit economically from the service MIDATA provides to researchers and companies who want to access the data of cooperative members. The cooperative does not distribute any income to members: the users’ benefit consists in improved services instead, together with the possibility of controlling the secondary uses of their data^5^. Also, the members are not permitted to exchange their data for money individually through the platform. Revenue gained from user fees is employed to cover operational costs, repay loans, or improve the platform service itself. Private investors cannot invest or buy economic shares in the cooperative: only nominal “participation shares”, which cannot be sold, and entitling to no economic rewards, are permitted^5^.

MIDATA is currently financed by research grants from a Swiss university, loans by private citizens, and scientific research grants on a project-by-project basis (Ernst Hafen, personal communication, 17 May 2016). Beyond the start up phase, MIDATA plans to achieve economic sustainability from fees paid for cooperative members for its personal data secure storage service and fees paid by companies and research institutions accessing the data stored by cooperative members (which members agree to make accessible) (Ernst Hafen, personal communication, 17 May 2016). As with many other technological start up companies, whether this economic model is sustainable in the long run, remains to be seen.

### ePGA

ePGA is a Greece-based platform and aims to translate pharmacogenomics information into a clinically meaningful format. It is targeted at three user groups: health professionals, biomedical researchers and individuals. As the website states, the aim of the platform is to provide a “‘one stop’ web-based platform, for pharmacogenomics knowledge recording, processing, assimilation and sharing”.

The data on the platform are comprised of information from established genomic databases and subsequently uploaded genomic profiles and phenotypes by the three groups of platform users (researchers, clinicians or interested individuals), creating a constantly evolving knowledge base. It relies on stakeholders to upload genotype information into the database. While researchers can query the database with regard to their research questions, individual patients and healthcare professionals can also obtain individualised advice on the basis of the genetic information submitted. The platform aims to provide practical advice for clinicians who lack training in pharmacogenomics, with regard to translating patient’s genomic information into concrete clinical advice, such as recommending adjustments to standard dosages. Patients or healthy individuals may also upload their genomic profile and can receive tips regarding the pharmacogenomic features of their profile. However, the platform emphasises that it will require clinicians’ advice for clinical decisions on the basis of this information.

In relation to scientific value, the development of the platform has been driven by genomic scientists on the basis of well established pharmacogenomics findings and databases (e.g., PharmGKB) and the platform is designed to meet high standards of scientific accuracy. The database incentivises contributions by established genomic researchers through a system of ‘microattribution’, so that the data they have contributed will be acknowledged in publications that utilise the data (Patrinos et al. 2012). This system aims to achieve a research-led expansion of the database. It is unclear from the available information which phenotypes will be included, particularly whether it exclusively relates to metaboliser status. If other information is to be included, and if individual users or researchers or clinicians are given the opportunity to provide such information, this might lead to variation in the scientific quality of the data. The current level of accessibility, with no access barriers for researchers, allows for optimal exploitation of this data for research purposes. However, unrestricted access might also raise potential ethical concerns. As the platform is still in its initial stages, it will remain to be seen what level of utilisation it will attract, which will determine its ultimate scientific utility.

To ensure user protection, individual users create a password-protected profile when uploading their genomic profile and other information. The exact type and extent of further security features is still unclear from the information available to the public. Potamios et al. (2014) highlight the importance of security and privacy issues for genomics platforms in their conceptual paper, but to date, no dedicated information on the platform’s security features appears to be available on the website. The authors also discuss the potential integration with personal health data, but again it is unclear whether and to what extent additional personal and health information may be stored in conjunction with the genomic information.

The platform aims to achieve a high level of user agency by directing users to access the platform through one of three services which are tailored for members of three stakeholder groups, based on their particular pharmacogenomics related interests. It is evident that the platform is still under development and that the users it primarily targets currently are researchers and health professionals, rather than non-scientific users. Still, individuals also have the opportunity of accessing the platform for analysis of their individual profile (George Patrinos, personal communication, May 5, 2016). Its transparency for these users appears limited and the platform does not offer instructions, examples, or more easily accessible information required for the informed consent process, such as lay information on what genomic and phenotypic data can be uploaded and queried. Nor does it describe what happens to the uploaded information, any potential privacy and security concerns, or the types secondary uses of the data by other parties.

One of the core concerns of the public with regard to the assessment of the trustworthiness of genomics initiatives has been the role of private partners (Vayena and Gasser 2016; Dove and Özdemir 2015). In the case of the ePGA, its development and maintenance have been funded by public grants, while no commercialisation is envisaged. This is likely to increase trustworthiness for users, as their personal information will not be exploited for financial gain. Additional trustworthiness concerns may be linked to data access modalities, data security and anonymity, on which little information is currently available.

The potential benefits to users of this platform are well defined and achievable, provided the platform proves to be user-friendly in its everyday operation. The platform gives health professionals instructions on how to adjust their prescriptions to their patients’ pharmacogenomic features on the basis of established and evolving pharmacogenomic knowledge. Similarly, individuals have the opportunity to obtain information on their own pharmacogenomic characteristics, which they can bring to the attention of their healthcare providers. The availability of this information is likely to translate directly into benefit to patients, by helping optimise dosage and preventing side-effects resulting from over-dosing or lack of effectiveness resulting from under-dosing. The open accessibility of a platform dedicated to pharmacogenomic knowledge is also likely to be of benefit to the pharmacogenomic research community, especially given the creation of the platform as a research-led initiative. This has the potential to impact on healthcare practices more broadly with regard to the use of pharmacogenomic information in the clinic.

The platform has been publicly funded through a number of international and national grants.^6^ Its long-term sustainability is dependent on the continued availability of grants or the future development of an alternative funding model. The platform coordinators are confident about the viability of the current funding approach for the nearer future, but aim to find longer term solutions once the platform becomes more established (George Patrinos, personal communication, May 11 2016).

### Platform for engaging everyone responsibly (PEER)

PEER is a project run by the Genetic Alliance. Genetic Alliance is a network of more than 1200 diseases advocacy organisations based in the United States. PEER is based on the concept of being as user-friendly and personalised as possible. The set-up of sites for individual groups is simplified and easy to customise and the appearance of the interface can be edited. Similarly, the services that individual users can access have many customisation options. In PEER it is possible to share not only health data, but also bio-specimens through the established network of participants in the platform, aiming to overcome the physical limits of ordinary biobanks.

In relation to scientific value, PEER users are responsible for the quality and amount of information they upload. The contribution of data for studies is dependent on user preferences, since the users choose what kind of data they will share, and researchers gain access to their information on the basis of these preferences.

User protection is addressed through accountability, which is one of the principles of PEER. All virtual processes such as edits, searches and users’ privacy settings are recorded, allowing PEER to track and investigate any suspicious uses of information or unauthorised changes. This data will be returned to the individual user in the form of a system report. Their privacy policy clearly identifies the responsibilities of Privacy Access and the Global Alliance assuring transparency.

This platform allows user agency through a significant degree of control over the terms of sharing health data and biological samples. The users have the authority to decide if researchers, relatives or doctors can see their data, and can also decide whether other users will have access to all, or just components of the data uploaded. Even though they belong to a community using PEER, all the data is shared according to users’ individual preferences. Other additional filters regarding the use of data can be determined by the wider community the user belongs to, while the platform uses a specific technology and software developed by the Private Access company to combine the individual and the community settings for sharing data. The data are stored in the PEER platform and users can edit them anytime. Accordingly, consent here seems to be similar to a refined broad consent (Kaye et al. 2009): the users have more options than just to share everything or nothing. However, once consent is given, users cannot control the destiny of their data unless they withdraw consent. The user’s agency is also facilitated through additional strategies, such as through constructing and joining new communities within the PEER platform, which allows their data to be aggregated with a pool of individuals with the same or a similar disease. The platform also aims to expand its reach to other communities by including additional languages, the first of which will be Spanish (Sharon Terry, personal communication, 2 March, 2016).

Trust is a core guiding concept for PEER. Health data and biological samples can be interchanged and shared in “an environment that provides the look and feel of familiar trusted communities” (PEER website). The extensive possibilities for users to customise their sharing preferences allow a significant degree of control over what happens to their data. The software also makes it possible to give feedback to the users on who is accessing their data, what is likely to increase trust.

PEER is particularly interested in accelerating progress in medical research by connecting participants who can share their data on a comprehensive platform across different diseases. The platform focuses on benefit for the common good, rather than benefit to individual users. This is the same principle used for the community’s creation. User benefits consist of possibilities for networking, although scientific or clinical benefits are mostly defined in future terms, such as advances in medical research enabled through the use of information on the platform or through research recruitment via the platform. Such advances in knowledge have the potential to benefit individuals in the future by identifying underlying genomic features of diseases and potentially develop new treatments on this basis.

The sustainability of the platform is ensured by funding from a wide range of public and private sources obtained by the platform. Crowdsourcing is one of the sources of funding for the platform. PEER has also been involved in a number of major initiatives, some of which were associated with substantial funding.^7^

## Comparison of online health data sharing platforms

In this article we have assessed five data sharing platforms with regard to their respective innovative approaches to the sharing of personal health data. We analysed these platforms in light of the core values we considered to be critical for the ethical sharing of data using ICT platforms. It was evident from the analysis that the platforms provide their users with substantially different ways of engaging with their own health information. Implicit in these different modes of engagement is variation in the weighting attributed to each of the values. While sharing some commonalities, each platform has its own value profile, with some values emphasised more strongly and other values less prominently underscored.

Data sharing is generally based on the scientific rationale of openness. Openness here means that platforms promote the scientific collaborations in an atmosphere of scientific transparency, which also implies public availability of scientific data (see also European Comission, 2013). All the platforms reviewed endorse the scientific benefits of data sharing. However, the scientific rationale is embedded differently in these platforms. For all platforms considered here, scientific values coexist with the realisation of other values, such as benefits to individual users. What became particularly evident in the analysis of the platforms was the different understandings of the value of science implicit in these platforms: while a platform such as ePGA appears to be built on a traditional understanding of expert-led science, a platform such as PEER highlights a more participant-led approach to science. For some of these platforms, data sharing for scientific purposes is not presented as the primary purpose of the platform for its users. Taltioni provides a wide range of health management functionalities and appears to emphasize those functionalities more prominently to users than the data sharing aspect of the platform; it is effectively a platform for other data sharing platforms. Both Healthbank and MIDATA provide a service that can be used independently of any engagement in data sharing, making data sharing for scientific purposes optional for participants. One difference between MIDATA and Healthbank is that the latter is a health data exchange platform, while MIDATA is designed to be able to host and provide accessibility to research data of any kind.

All platforms acknowledge the importance of user protection and gaining informed consent from users for the use of their information. However, the approaches to user protection and the prominence given to user protection in platform communications differ between platforms. Not all platforms have an explicit privacy policy. ePGA shows the least transparency with regard to privacy, which is probably due to its current primary focus being on healthcare professionals and researchers, as opposed to individual users. The common concept of privacy as control of information is used by Healthbank, MIDATA and PEER, rather than considering that ensuring privacy requires information to be protected in a type of vault which is externally inaccessible.

MIDATA and Healthbank seem to emphasise the technical side of data security most explicitly. They employ encryption techniques and any data that is not explicitly marked as shareable is securely encrypted. MIDATA is in the process of making the platform software openly available in order to ensure transparency. PEER emphasises that an audit trail of any activity relating to personal data on the system can be made transparent to the users so as to identify any unauthorised activities, a feature it also shares with MIDATA. On the other hand, Healthbank emphasises that they do not employ cookies to track user activity. For those platforms that make data available to private sector partners, the privacy policies deserve particular attention. While Taltioni, MIDATA and Healthbank either share, or allow each individual user to share anonymised data, it is not entirely clear whether particular measures beyond anonymisation or end-to-end encryption are in place to achieve privacy.

In fact, a complete anonymisation is a target difficult to achieve in the current scenario of online data sharing (Shabani and Borry 2015), what could be acknowledged and accepted within certain limits (Snell et al. 2012). Indeed, a minimum level of compliance with data sharing risks is necessary to enable the scientific value of the platforms. To overcome this eventual limitation, an investment in transparency particularly in providing information to users about the risks of data sharing and respect users agency and their decision of how and to what extent they want to be involved should be promoted.

Particular importance is placed on the facilitation of user agency in most of these platforms. Interestingly, the platforms focus on quite different aspects of user agency. ePGA allows users to access pharmacogenomic information about themselves – information that they can use to alert their healthcare professionals to specific pharmacogenomic features, thereby allowing them an active role in fine-tuning their treatment. Taltioni provides a high degree of potential customisation through the provision of a wide range of apps that users have the option of using. PEER has particular strengths regarding its provision of an accessible, easy to use, customisable and transparent platform. It provides several helpful resources to support users to understand the platform and make meaningful decisions with regard to how they wish to use it. PEER, MIDATA and Healthbank all pay significant attention to the control that the user has over their sharing modalities and provide a variety of possibilities for personal customisation. Healthbank, MIDATA, and PEER explicitly frame users as owners of their data allowing them to decide what, how, and with whom to share data and empowering the individual data owner to make fine-grained decisions about data sharing through an IT interface. In addition, in PEER, users can make decisions about sharing biological samples. In both Healthbank and MIDATA, it is the individual who uploads the data who decides what, and with whom, to share data. In Taltioni, the platform is allowed to use anonymised data if it has been authorised by the user. Non-anonymised information will only be shared if users have given permission. In Healthbank and MIDATA, only data explicitly selected for sharing by users/cooperative members are exported in an anonymised form. While in Healthbank such data can be made accessible for an economic return to the data owner, this is not the case in MIDATA.

Additional forms of user agency are evident in the models underlying Healthbank and MIDATA. In Healthbank, the potential of persons to leverage their personal data for profit is highlighted and each Healthbank user can sell access to their own data individually. MIDATA embodies a particularly strong sense of the collective by providing a basic platform, which enables the user to control data storage, and sharing, while allowing the community of users to develop platform-compatible software applications to access the data that individuals have unlocked. In the case of MIDATA, a strong value-driven cooperative ethos is evident in the set-up, where user agency is strongly linked to providing opportunities of genuine collective decision-making and joint ownership. This also leads, however, to limitations of what can be done with the data by the cooperative bylaws and the requirement of ethics board approval for any exchanges involving health data. The cooperative bylaws can be changed by users collectively, but only through collective agency, rather than individual autonomy.

The elements of user agency contribute particularly to the perceived trustworthiness of these platforms. PEER’s name, *Platform for Engaging Everyone Responsibly* clearly exemplifies this self-understanding. Healthbank, MIDATA and PEER present their platform as embodying trustworthiness, by facilitating user control in different ways. Healthbank focuses specifically on the availability of individualised control and high levels of data security. MIDATA instead focuses on a collective governance model and the absence of commercial actors. PEER facilitates the creation of activist communities to which individuals can affiliate themselves, on the basis of a transparent and customisable interface. In contrast, Taltioni’s trustworthiness is based on its endorsement by the Finnish state and its integration with the Finnish healthcare system, as well as users’ ability to decide which platforms to use and which data to share. ePGA’s presents its trustworthiness as predominantly scientifically based, given the primary emphasis on its scientific grounding and the complete absence of private sector interests. A number of commentators have suggested that the emphasis on agency through user-centredness in data sharing reflects an ethically desirable shift towards the empowerment of patients and research participants (Corrigan and Tutton 2006). This move can be seen in the implementation of platforms where dynamic consent is used to solve the traditional problem of consent (Kaye et al. 2011), as well as in cases where patient organisations take the lead in implementing data sharing initiatives, such as PEER (Novas 2007). Although Lupton (2014) has been critical of this transformation, the platforms that we have examined greatly emphasise the role of users in deciding and controlling (to a large degree) which information about themselves is shared and how it is done. User-centredness, however, poses problems for all platforms because if users decide not to contribute and continue to add data then the possibility of a sustained platform becomes problematic. Despite the support of the Finnish state, Taltioni is still very reliant on users to continue to use the services which are provided. It is unclear if it will succeed in its goal due to the low rates of user interaction and the difficulty that users may have in integrating it into their daily lives.

Benefits and benefit sharing are conceptualised differently in the five platforms. In the ePGA the benefit is envisaged as an immediate health benefit arising from improved prescribing, as well as a more indirect health benefit potentially arising from the improvement in the pharmacogenomic knowledge base through extensive data sharing. The features of the platform are immediately useful to different user groups and can be easily integrated in healthcare practice, not least through translation into higher quality services from the perspective of healthcare professionals using the platform (Lakiotaki et al. 2014). In Taltioni, the array of health management apps linked to the platform also constitute an obvious benefit to users, with the promise of achieving better health through access to new health management opportunities on the platform, but whether users will continue to use the services is very unclear. In Healthbank, the benefit foregrounded in the platform’s communications is the potential return of their investment to members in the form of valuable results or monetary benefits from the sale of their data. The profit would be shared among participants in each venture, depending on the value of the user’s data. While MIDATA also aims to achieve benefits for its members, the use of its revenue will be determined by the collective and is meant to contribute to the common good, particularly through ensuring sustainability and continuous improvement of the platform. Members are expected to derive a benefit from using the platform itself, for its data security features, the opportunities for controlled sharing, and also for the additional services that will be successively developed on the platform. The PEER platform promises benefits particularly in relation to return of research results and the potential to gain knowledge about their specific conditions by harnessing a larger collective for research on diseases than usually possible with traditional methods. Given its organisation in community structures, it may be argued that community benefit should be considered a real benefit for individuals who are part of these communities. However, where no immediate tangible benefits arise and any practical impact is merely projected for the future, one should question how real such benefits are and whether the data sharing reflects what Lupton (2014) has argued to be a new form of patient labour (see also Prainsack 2014b).

The platforms show very different approaches to the question of sustainability. As most of the platforms are fairly recent or still partly under development, it is difficult to assess their long term sustainability. Taltioni is supported by the Finnish government, but also involves private sector parties. How this cooperation will function in the longer term still remains to be seen. The lack of up-take by users will result in it not being supported by the State or by companies in the long-term. The PEER platform is probably the most well-established platform and has received substantial investment from a variety of sources. Its combination of crowdsourcing and grant funding appears to be a functioning model. With regard to Healthbank and MIDATA, whether health data proves to be a sufficiently valuable commodity to make their model economically sustainable in the long term remains to be seen. The ePGA still needs to develop a long term funding model.

Implicit in the organisation of these platforms and the values that they represent are particular understandings of health. The ePGA probably endorses the most traditional model of health, where the contribution of the platform is to optimise use of existing pharmacogenomic information, with immediate translation into increasing the accuracy of professionals’ prescription decisions. By focusing on linking research and healthcare advice, ePGA appears to endorse an evidence-based, knowledge-focused model of healthcare. However, individuals are also actively encouraged to obtain relevant information to achieve higher quality care (O’Riordan 2013). In contrast, Taltioni conveys to the user that health is a personal enterprise, and not merely the result of the passive receipt of evidence-based professional services. Health is more than routine health exams and controls; it is also related to well-being and individuals are invited to engage in healthy lifestyles activities (Lupton and Petersen 1996). But with this invitation also comes the expectation that users take responsibility for their health, potentially as an all-encompassing project. Conveying yet another model, Healthbank envisions health and illness as a matter of economic investment where the health data can be used to achieve profit: individuals and their records are themselves subjected to generation of profit.

MIDATA instead places its emphasis on health as a collective endeavour closely linked to the achievement of citizen empowerment. It appeals to members’ altruism by presenting the opportunity to benefit society as and of itself as reason to join, in much the same way as people in many European countries are not allowed to receive money for their blood donations. Similarly, the PEER platform connects the construction of health and illness directly with the notion of community. Its disease-based communities are understood as having the potential to drive significant advances for the common good (Novas 2007), outside of traditional models of healthcare and research, and to shape the future of medical research especially for the underserved area of rare diseases.

## Conclusion

We have illustrated how different approaches to data sharing in innovative online platforms are underscored by a set of values. The platforms we analysed show substantial differences in how they embody these values. The particular value profile of a platform influences the design and functioning of the platform, including how individuals can interact with the web interface, which data are shared, and under which circumstances. How health information is being shared and distributed in online platforms is never value neutral, and the identification of ethical considerations requires careful attention to particular features of individual platforms. Further empirical research and analysis is needed in order to comprehend the impact and the consequences of sharing health data through online platforms. It should also be noted that data sharing platforms contain many trade offs depending on the nature and scope of the platform. Nationally based platforms, such as Taltioni, are limited in their scope within the state, but provide the population with the opportunity to participate in a national initiative. Other networks, such as the PEER network, are global in scope, but may suffer due to the lack of consistency or homogeneity of participation, leading to problems with data reliability.

All platforms, however, share this problem to some extent insofar as they lack complete coverage of the population and therefore incur data reliability concerns. Despite political efforts to achieve larger scale data sharing across Europe, there remains a lack of coordination within the European context. Still, there is a significant potential for data sharing to be further developed in a socially robust and morally responsible way across Europe. The data sharing platforms discussed in this paper illustrate the likely complexity and potential diversity of future innovations in the field.

## Appendix

**Table 3:** List of initial online platforms for sharing health data

Platform	WWW address
Taltioni	http://taltioni.fi/
Healthbank	https://www.healthbank.coop/
ePGA	http://www.epga.gr/
PEER Network	http://peerplatform.org/
PatientsLikeMe	https://www.patientslikeme.com/
Genomera	Not available
Personal Genome Project	http://personalgenomes.org/
DNA Land	https://dna.land/
Sundhed.dk	https://www.sundhed.dk/
23andMe	https://www.23andme.com/
DNA.bits	http://socialm1.wix.com/dnabits
Interpretome	http://interpretome.com/
MIDATA	https://www.midata.coop/
ONCO-i2b2	Not available
TranSMART	http://transmartfoundation.org/
FINDbase	www.FINDbase.org
KORA	http://www.helmholtz-muenchen.de/kora

## Abbreviations

EU: European Union
GA4GH: Global Alliance for Genomics and Health
ICT: Information and Communication Technologies
IT: Information and Technology
PEER: Platform for Engaging Everyone Responsibly

## Footnotes

1 COST Action CHIP ME – Citizen’s Health through public-private initiatives:Public Health, Market and Ethical Perspectives (COST Action IS 1303) is an interdisciplinary network of researchers from all over Europe that meet on a regular basis to discuss issues relating to the intersection between public-private partnerships, genetics/genomics and ICT.

2 Taltioni Data Sharing Policy; available at: http://taltioni.fi/wp-content/uploads/2014/06/Taltioni-tietosuojaseloste.pdf

3 Healthbank Innovation AG, Baar. Accessed May 2016 at: http://www.moneyhouse.ch/u/healthbank_innovation_ag_CH-170.3.039.845-6.htm.

4 Healthbank FAQ section; available at: https://www.healthbank.coop/faq/

5 For more information see MIDATA Cooperative Statute 2017 (valid since 04 03 2017), unofficial English translation, forthcoming in MIDATA website.

6 ePGA was funded by European Commission (Grant: FP7–305444) and Greek National Secretariat of Research and Technology (Grant: ΠΔΕ11_046).

7 PEER is funded by The White House Initiative on Precision Medicine, the National Patient-Centered Clinical Research Network (PCORnet), the Patient-Centered Outcomes Research Institute (PCORI), the FDA’s Patient-Focused Drug Development initiative (PFDD) for which PEER was supported with a grant by the Pharmaceutical Research and Manufacturers of America (PhRMA) and the Robert Wood Johnson Foundation.
